# Risk and Prognostic Factors for BRAF^V600E^ Mutations in Papillary Thyroid Carcinoma

**DOI:** 10.1155/2022/9959649

**Published:** 2022-05-18

**Authors:** Xiaojing Wei, Xiaodong Wang, Jie Xiong, Chen Li, Yixuan Liao, Yongjun Zhu, Jingxin Mao

**Affiliations:** ^1^Chongqing Jiaotong University Hospital, Chongqing 400074, China; ^2^Chongqing Medical and Pharmaceutical College, Chongqing 400030, China; ^3^Department of Pharmacy, Ministry of Education Key Laboratory of Child Development and Disorders, National Clinical Research Center for Child Health and Disorders/Chongqing Key Laboratory of Pediatrics/Children's Hospital of Chongqing Medical University, Chongqing 400014, China; ^4^Department of Biology, Chemistry, Pharmacy, Free University of Berlin, Berlin 14195, Germany; ^5^College of Pharmaceutical Sciences, Southwest University, Chongqing 400715, China; ^6^The Orthopedics department of Ninth People's Hospital of Chongqing, Chongqing 400700, China

## Abstract

**Background:**

Over the past ten years, the incidence rate of papillary thyroid carcinoma (PTC) worldwide has been increasing rapidly year by year, with the incidence rate increasing 6% annually. PTC has become the malignant tumor with the highest growth rate in the world that fourteen PTC-related mutant genes have been identified. Whether the BRAF^V600E^ mutation related to more aggressive clinicopathologic features and worse outcome in PTC remains variable and controversial. We aim to investigate the risk factors that may predict the BRAF^V600E^ mutation potential of these lesions and new prevention strategies in PTC patients.

**Methods:**

A total of 9,908 papillary thyroid carcinoma patients with average 74.6% BRAF^V600E^ mutations were analyzed (RevMan 5.3 software) in this study. The PubMed, Embase, and ISI Web of Science databases were systematically searched for works published through December 15, 2021.

**Results:**

The following variables were associated with an increased risk of BRAF^V600E^ mutation in PTC patients: age ≥ 45 years (OR = 1.39, 95%CI = 1.21–1.60, *p* < 0.00001), male gender (OR = 1.13, 95%CI = 0.99–1.28, *p* = 0.06), multifocality (OR = 1.22, 95%CI = 1.07–1.40, *p* = 0.004), lymph node metastasis (OR = 1.33, 95%CI = 0.79–2.23, *p* = 0.28), extrathyroidal extension + (OR = 1.61, 95%CI = 1.06–2.44, *p* = 0.03), vascular invasion + (OR = 2.04, 95%CI = 1.32–3.15, *p* = 0.001), and tumor node metastasis stage (OR = 1.61, 95%CI = 1.38–1.88, *p* < 0.00001). In addition, tumor size (>1 cm) (OR = 0.51, 95%CI = 0.32–0.81, *p* = 0.005) and distant metastasis (OR = 0.69, 95%CI = 0.22–2.21, *p* = 0.54) had no association or risk with BRAF^V600E^ mutation in PTC patients.

**Conclusion:**

Our systematic review identified the following significant risk factors of BRAF^V600E^ mutation in PTC patients: age (≥45 years), gender (male), multifocality, lymph node metastasis, vascular invasion, extrathyroidal extension, and advanced tumor node metastasis stage (stages III and IV). Tumor size (>1 cm) and distant metastasis do not appear to be correlated with BRAF^V600E^ mutation in PTC patients.

## 1. Background

Thyroid cancer (TC) is the most common endocrine malignancy, with a relatively good prognosis after early diagnosis and treatment [1]. TC is usually classified into five different morphological groups which include papillary, follicular, medullary, poorly differentiated, and undifferentiated [2]. Nowadays, a combination of fine-needle aspiration (FNA) and ultrasound (US) is reliable to be used as a routine method for preoperative evaluation of thyroid [3]. There are benefits from the improvement of detection methods; the prevalence of TC is rising in recently years, and the most common subtype is papillary thyroid carcinoma (PTC) accounting for 80~85% [4]. In addition, the World Health Organization (WHO) defines tumors less than 1 cm as papillary thyroid microcarcinoma (PTMC) [5]. Although outstanding outcome and clinical indolence of papillary thyroid carcinoma patients (PTCs), aggressive clinical characteristics, and poor prognosis were also found in a small proportion of PTCs [6], it was reported that some PTCs are more aggressive with lymph node metastasis (LNM) and distant metastasis which may cause high mortality and poor prognosis [7]. Risk stratification is important to identify patients with a higher risk of recurrence, so more aggressive management and monitoring can be implemented [8]. Therefore, various risk stratification methods have been used to treat PTC patients properly and reasonably. Molecular markers for predicting PTC have been widely used to improve the risk stratification of PTCs in recent years [9]. Identifying molecular markers that can recognize these aggressive tumors, especially at the preoperative stage, is very useful for guiding the clinical treatment of PTCs [10]. B-type Raf kinase (BRAF) is a cytoplasmic protein kinase, a major subtype of Raf kinase, which triggers tumorigenesis by activating the MAPK pathway [11]. The pathogenic PTC mutations include BRAF^V600E^ mutation, RET/PTC rearrangement, and/or RAS mutation for most of patients [12]. The BRAF^V600E^ mutation frequently and specifically occurred in PTCs with a frequency of 25~82.3% while it is usually absent in other types of thyroid tumors [13]. In addition, BRAF^V600E^ mutations commonly occur in advanced PTC, which may enhance the ability of BRAF-mutant cells to proliferate into cancer cells [14]. Whether the BRAF^V600E^ mutations related to more aggressive clinicopathologic features and worse outcome remains variable and controversial. Hence, we aim to explore the clinicopathological significance of BRAF^V600E^ mutations in patients with PTC in this meta-analysis. Moreover, the results of our meta-analysis may also be helpful to assist the surgeons to choose the best surgical managements, such as whether the prophylactic central neck dissection (PCND) is needed and the risk stratification after PTCs.

## 2. Methods

We followed the methods of Mao et al. [15].

### 2.1. Search Strategy

The protocol of this overview was registered on the International Prospective Register of Systematic Reviews (PROSPERO) with registration number CRD42021278949 (http://www.crd.york.ac.uk/PROSPERO). The relevant published articles including PubMed, Embase, and ISI Web of Science databases were used to identify until December 15, 2021. The following keywords were used in searching: “BRAF^V600E^ mutation OR BRAF mutation” AND “clinical characteristics OR prognostic factor OR risk factor” AND “papillary thyroid carcinoma OR PTC OR papillary thyroid microcarcinoma OR PTMC”. Relevant articles were used to broaden the search scope, and all retrieved studies, reviews, and conference abstracts were retrieved by the computer. If multiple published studies describe the same population, we extract only the most complete or recent one. Three authors independently completed the selection process and resolved the differences through discussion. In addition, the research strictly follows the recommendations of the preferred reporting items for systematic review and meta-analysis (PRISMA) reporting.

### 2.2. Selection Criteria

The selection strategy used the following criteria: (a) prospective or retrospective original studies; (b) English language studies; (c) pathological confirmation of PTC during or after operation; and (d) available data on PTC risk or prognostic factors and sufficient forms of data extraction to calculate the odds ratio (OR).

The following exclusion criteria were adapted to exclude studies from meta-analysis: (a) reviews, case reports, editorials, letters to editors, meetings, and conference records; (b) insufficient data (e.g., less than 30 patients in the studying or research); (c) research using big data (e.g., using SEER study data); and (d) studying period beyond 5 years.

### 2.3. Data Extraction

Three authors abstracted the following data from the included articles: first author, country, publication years, case number, number of BRAF^V600^ mutation, and PTC-related risk factors. Age, gender, multifocality, tumor size, vascular invasion, LNM, extrathyroidal extension (ETE), tumor node metastasis (TNM) stage, and distant metastasis were concluded in the risk factors of PTC patients. The Newcastle-Ottawa quality assessment scale (NOS) was used to assess the quality of the research. Any disagreements were resolved by a third investigator (JXM).

### 2.4. Statistical Analysis

Statistical analysis of all meta analyses was performed using Review Manager version 5.3 (Cochrane Collaboration, Oxford, UK). The magnitude of the effect of each study was calculated by the OR or the weighted mean difference (WMD) of the 95% confidence interval (CI) briefly. A *p* value of <0.05 was considered statistically significant unless otherwise specified. In addition, the heterogeneity was quantified using the *Q*-test and the *I*^2^ statistic. When *p* > 0.1 and *I*^2^ < 50%, a fixed-effects model was applied; otherwise, a random-effects model was used. The Begg funnel plot was used to analyze for potential publication bias.

## 3. Results

**Figure 1:**
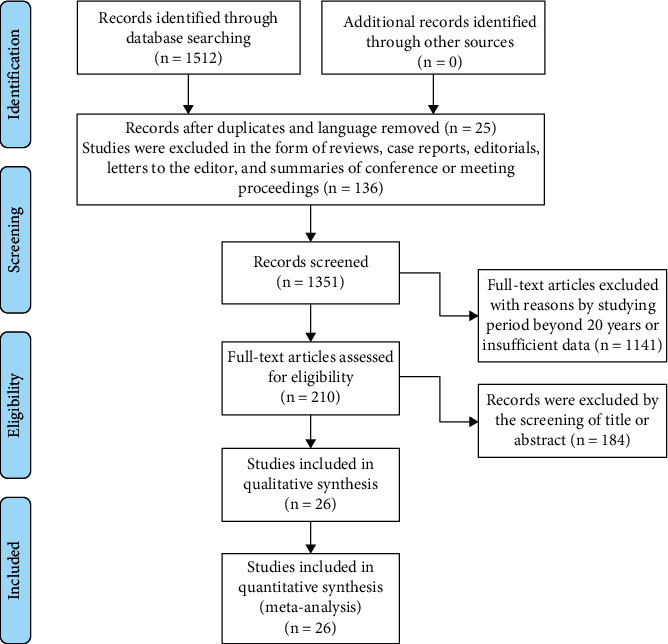
Flow chart of the study selection process.

After initially searching, a total of 1,512 studies were considered for inclusion in the meta-analysis. 25 records were excluded by language and duplicate; 136 records were excluded by the screening of reviews, letters, case reports, editorials, and meeting proceedings; 1141 records were excluded by using big data, studying period beyond 5 years, or insufficient data; 184 records were excluded by the screening of title or abstract. Finally, a total of 26 studies that met our selection criteria were included in our meta-analysis. The selection flowchart of research is presented in Figure 1. The basic characteristics of included studies and the associated prognostic factors examined are included in Table 1. In all the risk factor analyses, no significant asymmetry was found in Begg's funnel plot.

### 3.1. Prevalence of BRAF^V600E^ Mutation and Variables in PTCs

The prevalence of BRAF^V600E^-mutated population was a clinicopathological variable in a different study, ranging from 25.4% to 89.0%. Overall, BRAF^V600E^ mutation was confirmed among 7,395 patients of a total of 9,908 PTC patients in this systematic review and meta-analysis.

### 3.2. Risk Factors of BRAF^V600E^ Mutation in PTC Patients (Table 2)

#### 3.2.1. Age

**Figure 2:**
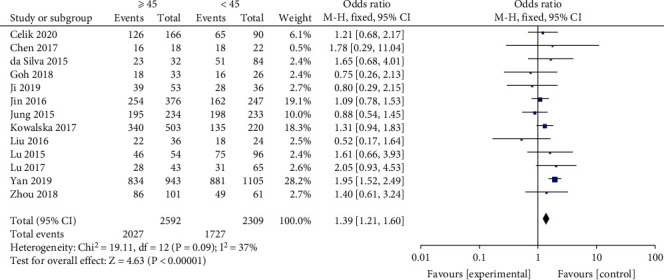
Forest plots of the association between age and BRAF^V600E^ mutation in papillary thyroid carcinoma (PTC) patients.

A fixed-effects model and input continuous data were selected using inverse variance method to calculate (*p* = 0.08, *I*^2^ = 36%). The results indicated that a significant association existed between BRAF^V600E^ mutation and age (age ≥ 45 years) in PTC patients (OR = 1.39, 95%CI = 1.21–1.60, *p* < 0.00001) (Figure 2).

#### 3.2.2. Gender

**Figure 3:**
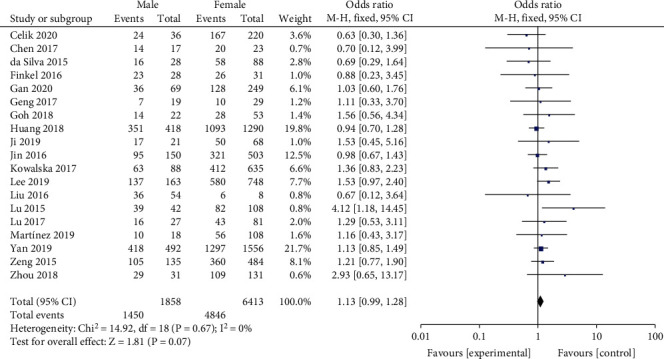
Forest plots of the association between gender and BRAF^V600E^ mutation in papillary thyroid carcinoma (PTC) patients.

A fixed-effects model was applied to analyze the data (*p* = 0.64, *I*^2^ = 0%). The prevalence of BRAF^V600E^ mutation in male PTC patients was relatively higher than that in female PTC patients (OR = 1.13, 95%CI = 0.99–1.28, *p* = 0.06 (Figure 3).

#### 3.2.3. Tumor Size

**Figure 4:**
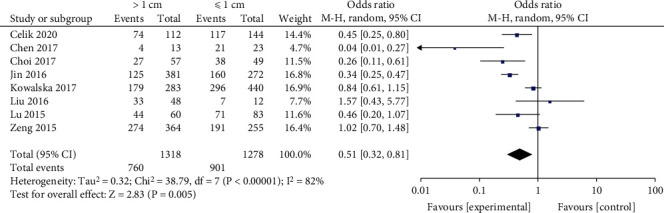
Forest plots of the association between tumor size and BRAF^V600E^ mutation in papillary thyroid carcinoma (PTC) patients.

A random-effects model and input continuous data were selected using inverse variance method to calculate (*p* < 0.00001, *I*^2^ = 82%). It was revealed that tumor size (≥1 cm) was not significantly associated with BRAF^V600E^ mutation in PTC patients (OR = 0.51, 95%CI = 0.32–0.81, *p* = 0.005) (Figure 4).

#### 3.2.4. Multifocality

**Figure 5:**
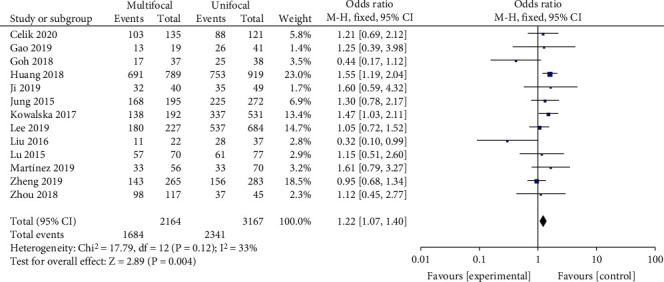
Forest plots of the association between multifocality and BRAF^V600E^ mutation in papillary thyroid carcinoma (PTC) patients.

A random-effects model was utilized to analyze the data (*p* < 0.12, *I*^2^ = 33%). It was demonstrated that tumor multifocality was associated with BRAF^V600E^ mutation in PTC patients (OR = 1.22, 95%CI = 1.07–1.40, *p* = 0.004) (Figure 5).

#### 3.2.5. Lymph Node Metastasis

**Figure 6:**
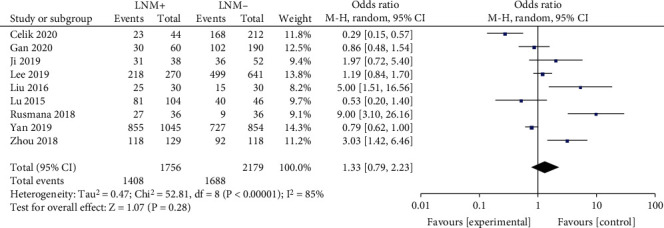
Forest plots of the association between LNM and BRAF^V600E^ mutation in papillary thyroid carcinoma (PTC) patients.

A fixed-effects model was utilized to analyze the data (*p* < 0.00001, *I*^2^ = 85%). It was revealed that LNM was significantly associated with BRAF^V600E^ mutation in PTC patients (OR = 1.33, 95%CI = 0.79–1.79, *p* = 0.28) (Figure 6).

#### 3.2.6. Extrathyroidal Extension

**Figure 7:**
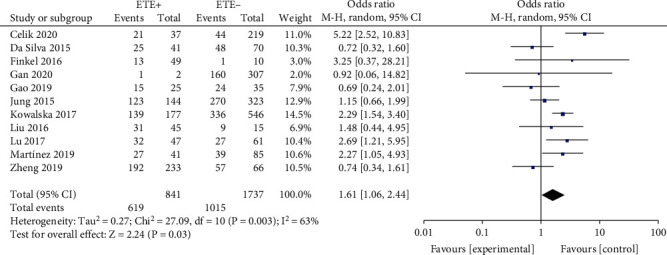
Forest plots of the association between ETE and BRAF^V600E^ mutation in papillary thyroid carcinoma (PTC) patients.

A random-effects model was used to analyze the data (*p* < 0.003, *I*^2^ = 63%). It was demonstrated that ETE was significantly related to a high rate of BRAF^V600E^ mutation in PTC patients (OR = 1.61, 95%CI = 1.06–2.44, *p* = 0.03) (Figure 7).

#### 3.2.7. Vascular Invasion

**Figure 8:**
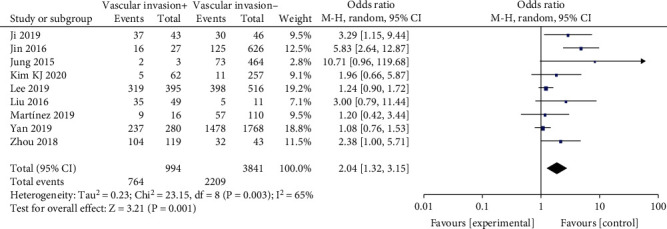
Forest plots of the association between vascular invasion and BRAF^V600E^ mutation in papillary thyroid carcinoma (PTC) patients.

A random-effects model was applied in the analysis involving vascular invasion (*p* = 0.003, *I*^2^ = 65%). It was indicated that vascular invasion exhibited a significantly high odds ratio for BRAF^V600E^ mutation in PTC patients (OR = 2.04, 95%CI = 1.32–3.15, *p* = 0.001) (Figure 8).

#### 3.2.8. Distant Metastasis

**Figure 9:**
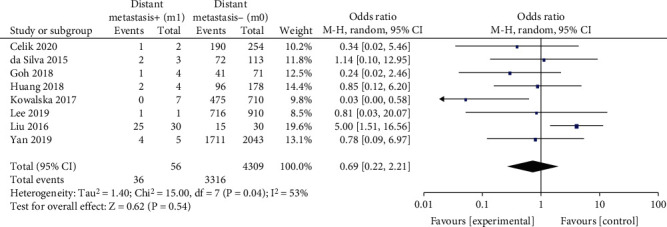
Forest plots of the association between distant metastasis and BRAF^V600E^ mutation in papillary thyroid carcinoma (PTC) patients.

A fixed-effects model was applied in the analysis (*p* = 0.04, *I*^2^ = 53%). It was found that distant metastasis was not associated with BRAF^V600E^ mutation in PTC patients (OR = 0.69, 95%CI = 0.22–2.21, *p* = 0.54) (Figure 9).

#### 3.2.9. Tumor Node Metastasis (TNM) Stage

**Figure 10:**
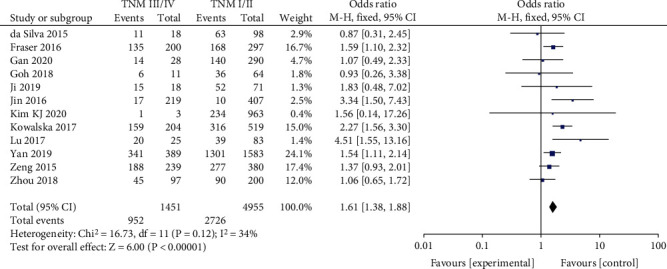
Forest plots of the association between TNM stage and BRAF^V600E^ mutation in papillary thyroid carcinoma (PTC) patients.

A fixed-effects model was utilized in the analysis (*p* = 0.12, *I*^2^ = 34%). It was demonstrated that TNM stage was significantly related to BRAF^V600E^ mutation in PTC patients (OR = 1.61, 95%CI = 1.38–1.88, *p* < 0.00001) (Figure 10).

#### 3.2.10. Publication Bias and Sensitivity Analysis

**Figure 11:**
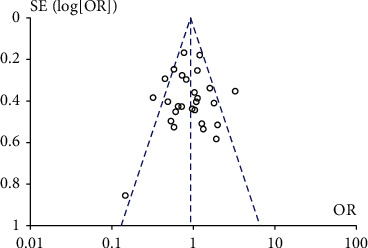
Funnel plot for publication bias analysis of the included articles.

Cochrane funnel plot was used to evaluate the publication bias, and no obvious asymmetric distribution was found in Figure 11 indicating that there was no publication bias.

## 4. Discussion

Although PTC is considered to be a malignant tumor, with good prognosis above 95% 10-year survival rate, it needed special attention and there is a need to watch out when vascular invasion, metastasis, or capsule invasion occur especially [16]. PTC also exhibits a biological characteristic of metastasizing to the surrounding neck lymph nodes easily, and some still develop recurrences which may be fatal [17]. One of the main clinical challenges in the treatment of PTCs is how to reliably classify patients who need active treatment to reduce the potential treatment-related morbidity and disease mortality, especially considering the lower overall mortality of PTCs [18]. In some researcher's opinion, PTC is also supposed to be a genetically driven disease. With the rapid development of translational medicine, the understanding of the pathogenesis and molecular spectrum of PTC has been greatly improved in recent years [19]. BRAF is one of the important biomarkers in human benign and malignant tumors, and most mutations affect BRAF^V600^ in exon 15 of the BRAF gene [20]. In addition, BRAF^V600E^ mutation is related to failure, recurrence, and mortality in PTC treatment which is considered an effective target for thyroid cancer [21]. However, some reports demonstrated that the BRAF^V600E^ mutations are not related to aggressive clinicopathologic features and worse outcome [22]. It remains variable and controversial. Therefore, on the one hand, the purpose of this meta-analysis was to determine whether BRAF^V600E^ mutations are associated with high-risk clinicopathological factors in PTC patients. On the other hand, it is necessary to explore the role of genetic events as reliable prognostic indicators in risk stratification and PTC management.

The association between age and BRAF^V600E^ mutation was analyzed in fourteen studies. It was demonstrated that age is a strong, continuous, and independent mortality risk factor in patients with BRAF^V600E^ mutation in patients with PTC [23]. Previous studies reported that age ≥ 45 years was association with the increased risk of BRAF^V600E^ mutations in PTC patients [24]. In the present meta-analysis, we found that the patients with old age (≥45 years) for PTC may have the increased risk of BRAF^V600E^ mutations in clinical practice (OR = 1.38).

The relationship between gender and BRAF^V600E^ mutation was analyzed in nineteen studies. Although the proportion of women and men in PTCs is 3 : 1, the rates of PTC-induced malignancies and mortality are higher in men [25]. In addition, it was reported that male sex is a robust independent risk factor for BRAF^V600E^ mutation in patients with PTCs [26]. Based on the analysis result, we also concluded that the gender of male was a significant risk factor for BRAF^V600E^ mutation in PTC patients (OR = 1.13).

Eight studies were analyzed for the correlation between tumor size and BRAF^V600E^ mutation in PTC patients. Generally speaking, tumor size is an important factor for TNM staging, and large tumor always exhibits aggressive characteristic [27]. It was revealed that BRAF^V600E^ mutation is associated with invasive tumor growth and tumor size (≥1 cm) in high-risk PTCs [28]. However, previous research also demonstrated that BRAF^V600E^ mutation was not correlated with tumor size (≥1 cm) in PTC patients [29]. In our meta-analysis, we found that tumor size ≥ 1 cm had no relation or risk with enough sources of variation BRAF^V600E^ mutations in PTC patients (OR = 0.51). Our finding was consistent with some reports in previous research. These conflicting findings between different studies may be due to different characteristics of the patients studied, including the sample sizes and proportions of different types of PTCs. In addition, different hospitals have different ultrasound equipment and different detection doctors. For the size of the tumor, human manipulation and subjective factors may have a greater impact on the final result.

Tumor multifocality is frequently observed in PTCs, but its prognostic value is controversial. It was reported that tumor multifocality is not considered to be an independent risk factor of BRAF^V600E^ mutation in PTC patients [30]. However, previous research also has demonstrated that BRAF^V600E^ mutation is closely related to tumor multifocality with poor prognosis and aggressively behavior in PTC patients [31]. Our results showed that BRAF^V600E^ mutation was related to multifocality in PTC patients which is analogous with previous research (OR = 1.22).

The association between LNM and BRAF^V600E^ mutation was analyzed in nine studies. LNM is commonly considered to be an important risk factor for recurrence and/or persistent disease and overall survival in PTCs [32]. In previous meta-analysis, it was reported that BRAF^V600E^ mutation is significantly related to LNM in PTC patients with poor outcome [33]. In the present meta-analysis, the prevalence of LNM was increased in PTC patients with BRAF^V600E^ mutation which means BRAF^V600E^ mutation was related to multifocality in PTC patients but with not enough sources of variation (OR = 1.33).

A total of eleven studies were analyzed for the correlation between ETE and BRAF^V600E^ mutation in PTC patients. The prognosis of the tumor is associated with the pathogenetic degree of ETE, and severely dilated extrathyroid disease is more severe than patients with histological examination showing local expansion [34]. A previous study also demonstrated that BRAF^V600E^ mutation is linked to the aggressive clinicopathological features especially ETE [35]. In our meta-analysis, there was significant association between ETE and BRAF^V600E^ mutation in PTC patients (OR = 1.61) which is similar with a previous study.

The relationship between vascular invasion and BRAF^V600E^ mutation in PTC patients was analyzed in nine studies. It was reported that vascular invasion of PTC patients is a sign of increased tendency of hematogenic invasion, which means finally a poorer prognosis [36]. In addition, it has been demonstrated that presence of tumor vascular invasion does not adversely influence biological behavior or survival of PTCs [37]. It was also revealed that BRAF^V600E^ mutation is more common in aggressive histological types of thyroid cancer and was likely to present in vascular invasion [38]. In the present meta-analysis, it was demonstrated that vascular invasion was significantly associated with BRAF^V600E^ mutation in PTC patients (OR = 2.04).

Distant metastasis is usually regarded as an indicator of the rapid development of PTCs. It has been demonstrated that BRAF^V600E^ mutation causes poorer prognosis including distant metastasis in PTC patients [39]. However, the previous study also showed that BRAF^V600E^ mutation is not related to the clinicopathological features such as the distant metastasis which affects the prognosis [40]. An interesting finding in the present meta-analysis is that the BRAF^V600E^ mutation had no relationship or risk with distant metastasis (OR = 0.69). A potential cause of this result may be different diagnoses of distant metastases in different countries and medical centers.

Twelve studies that were analyzed are associated with TNM stage and BRAF^V600E^ mutation in PTC patients. It was demonstrated that BRAF^V600E^ mutation is related to TNM stage, especially high stage which means poor prognosis [41]. In addition, it was also revealed that TNM stage is not related to BRAF^V600E^ mutation in PTC patients, although advanced TNM stage is more common among the BRAF^V600E^-positive patients [42]. In the present meta-analysis, we found the significant correlation between BRAF^V600E^ mutation and high stage (stages III and IV) in PTC with an odds ratio of 1.61.

Cohen et al. first discovered the existence of BRAF gene mutation in thyroid cancer in 2003; then, BRAF gene mutation is considered to be the most deeply studied gene in thyroid cancer molecular markers [43]. Mutations in the BRAF gene are particularly common in PTCs, with mutation rates ranging from 29% to 83% [44, 45] which is similar with us. In addition, BRAF is part of the mitogen-activated protein kinase (MAPK) signaling pathway, and the V600E mutation leads to the conversion of valine to glutamate, resulting in constitutive activation of BRAF, which leads to the transcription of genes involved in cell proliferation and promotes tumorigenesis, cell proliferation, and metastasis. BRAF mutation may also lead to decreased expression of iodine uptake genes in the thyroid gland, loss of human sodium iodide transport protein (NIS) gene expression, and misplaced distribution of NIS protein, causing some PTC patients to be resistant to radioactive iodine therapy and ultimately resulting in poor prognosis after treatment failure [46]. Previous studies have found that BRAF mutations are closely associated with aggressive pathological features of PTCs such as extrathyroidal invasion, lymph node metastasis, and later TNM staging [47, 48], even for PTMC [49]. A meta-analysis of 2470 PTCs showed that BRAF mutant had a higher recurrence rate than BRAF wild type (24.9% vs. 12.6%), and its sensitivity for predicting tumor recurrence was 65%, indicating that BRAF mutation is closely related to tumor recurrence [50]. Interestingly, it was reported that the mutation rate of BRAF in PTCs is relatively high, especially in Asian countries including South Korea, Japan, and China where the mutation rate can reach 68.7% [51]. In addition, previous studies have reported a positive association between active smoking and thyroid cancer risk which indicates that lifestyle may also influence the recurrence of PTCs [52]. Although the relationship between BRAF mutation and PTC clinicopathology and prognosis is controversial, it has been recognized as a “specific gene” of PTC; notably, the combination of thyroid nodule fine needle aspiration and BRAF mutation detection can significantly improve the detection rate of PTCs [53]. Recent clinical studies have reported that the selective BRAF inhibitor dabrafenib can activate cancer cells that do not uptake I131 to reexpress NIS and regain the function of I131 uptake, providing a new therapeutic hope for patients with BRAF-mutated I131-refractory metastatic PTCs [54].

Although the meta-analysis has investigated several clinical and pathological predictors of BRAF^V600E^ mutation risk that may help surgeons to choose appropriate treatment strategies and determine various risk stratification prognosis in PTC patients, there are still some limitations that exist in our study. Firstly, only 25 studies and recent five-year studies were included for predicting the risk of BRAF^V600E^ mutation and clinicopathologic features in PTC patients. Secondly, surgery performed by different physicians may also have influence on the accuracy of data analysis, even following the standard mode and operation quality. Thirdly, although PTC is also considered to be a genetically driven disease, there is only one molecular mechanism (BRAF^V600E^ mutations) that was discussed. It was revealed that coexistent TERT promoter and BRAF^V600E^ mutations may have a synergistic effect on clinical outcomes in PTCs [55]. Furthermore, it has been demonstrated that coexistence of BRAF^V600E^ and TERT promoter mutations are the most aggressive subgroup in PTC patients, while PTCs with BRAF or TERT alone are less aggressive [56]. Above all, to research those genetical mutations affiliated with PTC can help to stratify patients into distinct risk groups and better assess patients' outcome.

## 5. Conclusions

Taken together, this meta-analysis investigated the following risk factors and related links with BRAF^V600E^ mutation in PTC patients including age (≥45 years), gender (male), multifocality, LNM, vascular invasion, ETE, and advanced TNM stage (stages III and IV). Tumor size (≥1 cm) and distant metastasis were not correlated with BRAF^V600E^ mutation in PTC patients. In addition, based on the available evidence, BRAF^V600E^ mutation is significantly related to recurrence and PTC-related mortality as well. Therefore, molecular detection of BRAF^V600E^ mutation may help us clinically stratify the risk of PTCs and scientific management of patients.

## Figures and Tables

**Table 1:** Basic characteristics of included studies and the associated prognostic factors examined.

First author	Country	Publication years	Case number	No. of BRAF+ (%)	Age	Gender	Tumor size	Multifocality	LNM	ETE	Vascular invasion	Distant metastasis	TNM stage	NOS
Celik [57]	Turkey	2020	256	65 (25.4)	Y	Y	Y	Y	Y	Y	N	Y	N	9
Chen [58]	China	2017	40	34 (85.0)	Y	Y	Y	N	N	N	N	N	N	7
Choi [59]	Korea	2015	95	78 (82.1)	N	N	Y	N	N	N	N	N	N	6
da Silva [60]	Brasil	2015	116	74 (63.8)	Y	Y	N	N	N	Y	N	Y	Y	7
Finkel [61]	Israel	2016	59	49 (83.1)	N	Y	N	N	N	Y	N	N	N	6
Fraser [62]	Australia	2016	496	309 (62.3)	N	N	N	N	N	N	N	N	Y	5
Gan [63]	China	2020	475	239 (50.3)	N	Y	N	N	Y	Y	N	N	Y	7
Gao [64]	China	2019	60	39(65.0)	Y	Y	N	Y	N	Y	N	N	N	7
Goh [65]	Singapore	2018	75	42 (56.0)	Y	Y	N	Y	N	N	N	Y	Y	8
Huang [66]	China	2018	1708	1443 (84.5)	N	Y	N	Y	N	N	N	Y	N	6
Ji [67]	China	2019	89	67 (75.3)	Y	Y	N	Y	Y	N	Y	N	Y	8
Na [68]	China	2016	653	416 (63.7)	Y	Y	Y	N	N	N	Y	N	Y	7
Jung [69]	Korea	2015	302	265 (89.0)	Y	N	N	Y	N	Y	Y	N	N	7
Kim [70]	American	2020	241	215 (89.2)	N	N	N	N	N	N	Y	N	Y	6
Kowalska [71]	Poland	2017	723	475 (65.7)	Y	Y	Y	Y	N	Y	N	Y	Y	9
Lee [72]	Korea	2019	911	717 (78.8)	N	Y	N	Y	Y	N	Y	Y	N	8
Liu [73]	China	2016	60	40 (66.7)	Y	Y	Y	Y	Y	Y	Y	Y	N	9
Lu [74]	China	2015	150	121 (80.6)	Y	Y	Y	Y	Y	N	N	N	N	7
Lu [75]	China	2017	108	59 (54.6)	Y	Y	N	N	N	Y	N	N	Y	6
Martínez [76]	Chile	2019	126	66 (52.0)	N	Y	N	Y	N	Y	Y	N	N	7
Rusmana [77]	Indonesia	2018	36	21 (58.3)	N	N	N	N	Y	N	N	N	N	6
Yan [78]	China	2019	2048	1715 (83.7)	Y	Y	N	N	Y	N	Y	N	Y	8
Zeng [79]	China	2015	619	465 (75.1)	N	Y	Y	N	N	N	N	N	Y	7
Zheng [80]	China	2019	299	249 (83.3)	N	N	N	Y	N	Y	N	N	N	5
Zhou [81]	China	2018	163	135 (83.3)	Y	Y	N	Y	Y	N	Y	N	Y	8

BRAF^V600E^ + indicates the BRAF^V600E^ mutation; Y indicates that the study was evaluated for the correlatively prognostic factor; N indicates that the study was not evaluated for the correlatively prognostic factor.

**Table 2:** Risk factors for BRAF^V600E^ in PTC patients.

Risk factor	Pooled OR	95% CI	*p* value
Age (≥45 years)	1.39	1.21–1.60	<0.00001
Gender (male)	1.13	0.99–1.29	0.06
Tumor size	0.51	0.32–0.81	0.005
Multifocality (+)	1.22	1.07–1.40	0.004
Lymph node metastasis (+)	1.33	0.79–2.23	0.28
Extrathyroidal extension (+)	1.61	1.06–2.44	0.03
Vascular invasion (+)	2.04	1.32–3.15	0.001
Distant metastasis	0.69	0.22–2.21	0.54
TNM stage (+)	1.61	1.38–1.88	<0.00001

+ indicates the presented state.

## Data Availability

All data generated or analyzed in the study are included in this published article.

## Abbreviations

BRAF: B-type Raf kinase
CI: Confidence interval
ETE: Extrathyroidal extension
FNA: Fine-needle aspiration
LNM: Lymph node metastasis
NOS: Newcastle-Ottawa quality assessment scale
OR: Odds ratio
PTC: Papillary thyroid carcinoma
PTMC: Papillary thyroid microcarcinoma
PCND: Prophylactic central neck dissection
PRISMA: Preferred reporting items for systematic review and meta-analysis
TC: Thyroid cancer
TNM: Tumor node metastasis
US: Ultrasound
WHO: World Health Organization.

## Supplementary Materials

Supplementary Materials Supplementary Material PRISMA 2020 is an evidence-based minimum set of items for reporting in systematic reviews and meta-analyses. PRISMA primarily focuses on the reporting of reviews evaluating the effects of interventions but can also be used as a basis for reporting systematic reviews with objectives other than evaluating interventions (e.g., evaluating aetiology, prevalence, diagnosis, or prognosis). For authors: PRISMA is aimed at helping authors improve the reporting of systematic reviews and meta-analyses. For reviewers and editors: PRISMA may also be useful for critical appraisal of published systematic reviews, although it is not a quality assessment instrument to gauge the quality of a systematic review.
